# Broadband Ultra-Deep Sub-Diffraction-Limit Optical Focusing by Metallic Graded-Index (MGRIN) Lenses

**DOI:** 10.3390/nano7080221

**Published:** 2017-08-12

**Authors:** Yechuan Zhu, Weizheng Yuan, Hao Sun, Yiting Yu

**Affiliations:** 1 Key Laboratory of Micro/Nano Systems for Aerospace, Ministry of Education, Xi’an 710072, China; yechuanzhu_30@hotmail.de (Y.Z.); yuanwz@nwpu.edu.cn (W.Y.); sunhao0878@outlook.com (H.S.); 2 Key Laboratory of Micro- and Nano-Electro-Mechanical Systems of Shaanxi Province, Northwestern Polytechnical University, Xi’an 710072, China

**Keywords:** nanofocusing, coupled metallic waveguides, metallic graded-index lens, Hamiltonian optics

## Abstract

The development of techniques for efficiently confining energy in the visible and infrared spectral regions to the deep subwavelength spatial scale with dimensions as small as a few nanometers would have great significance for scientific research and engineering practices. Such an ability to manipulate light is impossible for conventional dielectric lenses due to the diffraction limit. Here, we propose a metallic graded-index (MGRIN) lens formed by an array of coupled metallic waveguides with identical nanoscale widths embedded by index-varying dielectrics to enable the optical nanofocusing. The focusing mechanism of the MGRIN lens is theoretically investigated based on Hamiltonian optics, which are verified by the finite-difference time-domain (FDTD) method. Numerical results reveal that an ultra-deep subwavelength focus of 8 nm (*λ*/500) with a long focal depth (1.93*λ*) and enhanced field intensity can be achieved. Moreover, the nanofocusing capability of the MGRIN lens without redesigning the structure can be well kept when the incident wavelength changes over a broad range from visible to infrared. Our design of optical nanofocusing shows great potential for use in nano-optics and nanotechnology.

## 1. Introduction

The spatial resolution of conventional optical devices is restricted by the diffraction to nearly half the operating wavelength, which greatly limits the performance of all the imaging and focusing systems that lie at the heart of modern biology, electronics, and optical integrated circuits [1,2,3]. In the past two decades, a great number of plasmonic devices have been designed to exceed the diffraction limit and manipulate the properties of light with nanometer-scale precision based on surface plasmon polaritons (SPPs) [4,5,6,7]. In particular, many efforts have been made in nanofocusing of light using various metallic structures [8], such as plasmonic nanoantennas and tapered metallic structures.

Plasmonic nanoantennas function as optical resonators that can support highly localized plasmon modes, thus leading to the strong enhancement of the localized field near the antennas [9]. Unlike nanofocusing through localized surface plasmon resonance, plasmonic nanofocusing by tapered metallic structures involves an increasing concentration of light energy into a nanoscale spatial region as the SPP mode propagates along a tapered plasmonic waveguide with gradually varying parameters [8]. The metallic structures for such nanofocusing mainly include tapered metal rods with rounded tips [10,11,12,13,14,15], metal wedges surrounded by dielectrics [16,17,18], tapered metal strips on dielectric substrates [19,20,21], tapered gaps formed by metal-insulator-metal (MIM) structures [22,23,24,25,26], and metallic V-grooves [27]. These open great opportunities for the development of a new class of nano-optical devices and techniques, such as optical signal processing in highly integrated nanophotonic circuits [28], nano-optical sensors [29], nanoimaging [30], and nanomechanics [31]. 

Completely different from the two typical ways of optical nanofocusing mentioned above, in this work, we propose a unique method for nanofocusing using a planar metallic graded-index (MGRIN) lens that is composed of multilayer coupled MIM waveguides. Numerical simulation confirms our design and demonstrates that nanofocusing with a long focal depth and enhanced field intensity can be achieved with the MGRIN lens for broadband frequencies from visible to infrared. This planar structure can find applications in wavelength division multiplexing, nanolithography, and highly integrated optical circuits.

## 2. MGRIN Lens Design

**Figure 1:**
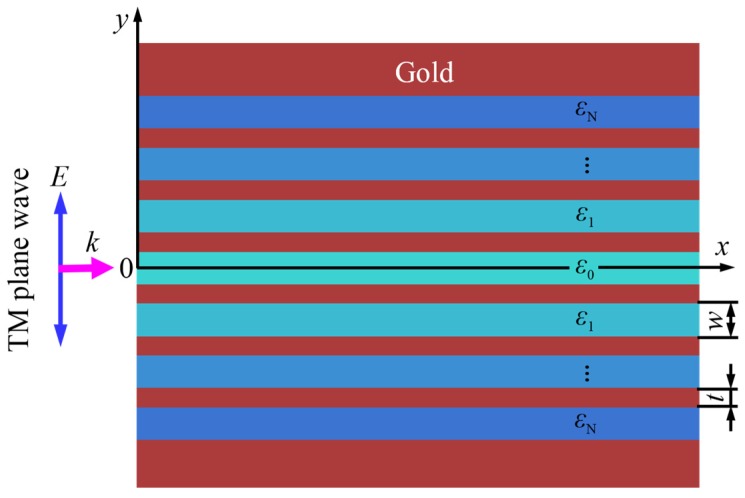
The schematic of a metallic graded-index (MGRIN) lens formed by coupled waveguides of uniform width and gold spacing under the normal incidence of a transverse magnetic plane wave. The structure is symmetric with respect to the central waveguide at *y* = 0, and *ε_n_* (0 ≤ *n* ≤ N) (*n* the integer with the values of 0, 1, 2… N) represents the permittivity of the dielectric in the waveguide *n*.

Metallic waveguides have an extremely distinctive advantage over dielectric structures in their support for deep subwavelength modes [32], thus enabling the manipulation of light at the nanoscale. Additionally, metallic waveguide arrays have been extensively proposed for optical focusing [33,34,35,36,37,38]. Particularly, in 2009, Verslegers et al. [39] designed an aperiodic metallic waveguide array using Hamiltonian optics and demonstrated a deep-subwavelength focus of 30 nm, as small as λ/100 (λ being the operating wavelength), in the central waveguide. However, in their design, the waveguide widths must increase from the two sides to the center, which limits the further reduction of the central waveguide width and the resulting nanofocusing capability. In contrast, an array of coupled metallic waveguides of the constant width and gold spacing but filled with different dielectrics is employed to form our MGRIN lens for the nanofocusing scheme, as shown in Figure 1.

In our design, the structure is symmetric with respect to the central waveguide at *y* = 0 and semi-infinite in the *z* direction, which is normally illuminated by a transverse magnetic (TM) plane wave. Because the permittivities of the dielectrics do not vary much between one waveguide and its adjacent counterpart, this structure can be locally considered periodic. Therefore, the consequent dispersion equation for a waveguide can be derived using the transfer matrix method [40]:

(1) $$\cos\;[k_{y}(w+t)]=\cos\;\left(k_{1}w\right)\cos\;\left(k_{2}t\right)\;-\frac{\varepsilon_{m}^{2}k_{1}^{2}+\varepsilon_{d}^{2}k_{2}^{2}}{2\varepsilon_{m}\varepsilon_{d}k_{1}k_{2}}\sin\;\left(k_{1}w\right)\;\sin\;\left(k_{2}t\right)$$

with *k*_1_ = (*ε_d_k*_0_^2^ − *k_x_*^2^)^1/2^ and *k*_2_ = (*ε_m_k*_0_^2^ − *k_x_*^2^)^1/2^, where *w* is the waveguide width, *t* is the metal spacing between two adjacent waveguides, *k*_0_ is the free space wave vector, and *k_x_* is the propagation wave vector in the *x* direction. *ε_m_* and *ε_d_* are the permittivities of the metal and dielectric material in the waveguide, respectively. Our nanofocusing structure is built based on the following propagation constant profile of the metallic waveguides along the *y* axis:

(2) $$\beta_{s}(y)=\;\beta_{s,0}(1+\;ay^{2})$$

where *β_s_* is the symmetric solution of *k_x_* in Equation (1) for *k_y_* = 0, and *β_s,_*_0_ is the corresponding value of the central waveguide. *a* is the gradient parameter. Since the effective refractive index of a waveguide is *n_e_* = *β_s_*/*k*_0_, Equation (2) can be transformed into the following:

(3) $$n_{e}(y)=n_{e,0}(1\;+ay^{2})$$

This quadratic effective index profile is similar to the design of a conventional graded-index lens [41], albeit the effective index increasing from the center to the side due to the negative refraction of a metallic waveguide array [42]. This is the reason why we consider this metallic waveguide array as an MGRIN lens. 

On the other hand, by choosing a proper parameter *b*, we can obtain the following approximation:

(4) $$\beta_{a}(y)\;=\beta_{a,0}(1\;+by^{2})$$

where *β_a_* is the antisymmetric solution of *k_x_* in Equation (1) for *k_y_* = *π*/(*w* + *t*), and *β_a,_*_0_ is the corresponding solution of the central waveguide. From Equations (2) and (4), the Hamiltonian can be deduced:

(5) $$H(y,k_{y})=k_{x}-\;\beta_{s,0}\;-a\beta_{s,0}y^{2}+\frac{1}{4}(\beta_{s,0}-\beta_{a,0}){\left(w+t\right)}^{2}k_{y}^{2}$$

Furthermore, the trajectory of a light ray in the structure can be solved by:

(6) $$\frac{dy}{dx}=\frac{\partial H(y,k_{y})}{\partial k_{y}},\frac{dk_{y}}{dx}=-\frac{\partial H(y,k_{y})}{\partial y}$$

From Equations (5) and (6), the analytical solution of a ray trajectory can be derived as follows:

(7) $$y(x)=C_{1}\sin(Gx)+C_{2}\cos(Gx)$$

with *G* = [(*β_a,_*_0_ − *β_s,_*_0_)*aβ_s,_*_0_]^1/2^(*w* + *t*), *C*_1_ and *C*_2_ are constants related to the position and angle of the incident ray. Assuming that at *x* = 0, the position is *y* = *y*_0_ and the corresponding slope is *y*’ = *y*_0_’, the above equation can be transformed into:

(8) $$y(x)=\frac{1}{G}{y^{\prime }}_{0}\sin(Gx)+y_{0}\cos(Gx)$$

In the case of normal incidence, since *y*_0_’ = 0, a ray trajectory can be further written as:

(9) $$y(x)=y_{0}\cos(Gx)$$

From Equation (9), it can be concluded that for a normally incident plane wave, all rays go through a cosine trajectory and consequently intersect at a focal length of *π*/(2*G*) when they propagate in the structure of an MGRIN lens. This is the self-focusing mechanism of our MGRIN lens.

## 3. Results and Discussion

**Figure 2:**
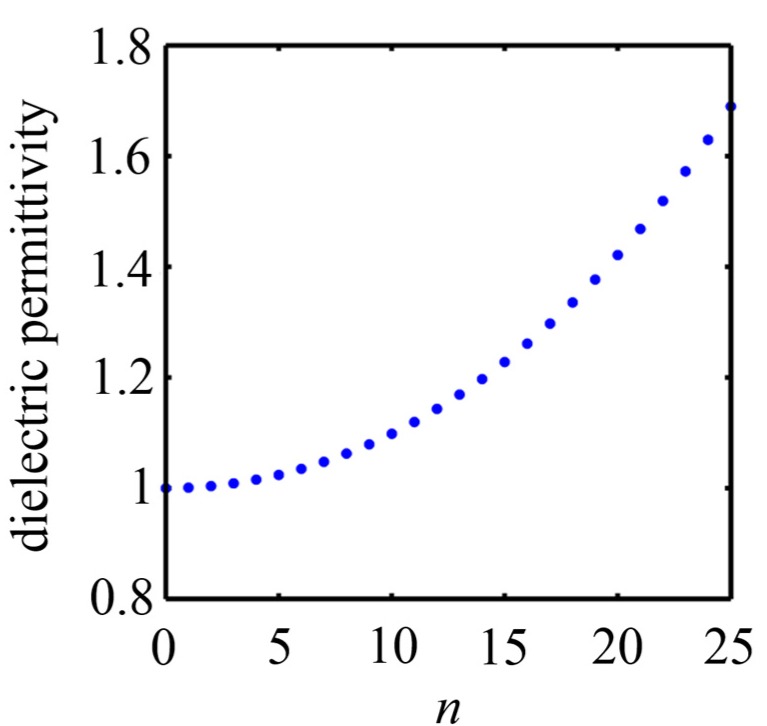
The required permittivity of the dielectric in the *n*th waveguide of an MGRIN lens working at *λ* = 1 μm. The MGRIN lens comprises a total of 51 waveguides and the dielectric constant increases from 1 at the center to 1.69 at the sides.

To demonstrate the focusing behavior, an MGRIN lens operating at a wavelength of 1 μm was designed with *ε*_0_ = 1 and *ε*_N_ = 1.69. At this wavelength, the permittivity of gold is *ε_m_* = −40.764 + 1.261*i* [43]. We considered a structure with a total of 51 waveguides that have the same width of 10 nm and are uniformly separated by 30 nm of gold. The required permittivity of the dielectric in the *n*th waveguide (0 ≤ *n* ≤ 25) is calculated by using Equations (1) and (2), as shown in Figure 2. The maximum variation in the dielectric constant between the adjacent waveguides is less than 0.06. Thus, it is reasonable to consider the structure to be locally periodic. 

By using the finite-difference time-domain (FDTD) simulation, the focusing performance of the designed MGRIN lens is analyzed. In simulations, the grid size is set to 1 nm in both *x* and *y* directions to model the fine features of the electromagnetic field in the structure. Perfectly matched layers as the absorbing boundary conditions are applied around the computational domain. The incident TM-polarized plane wave is defined by setting the electric field component *E_y_* with the amplitude of 1.

**Figure 3:**
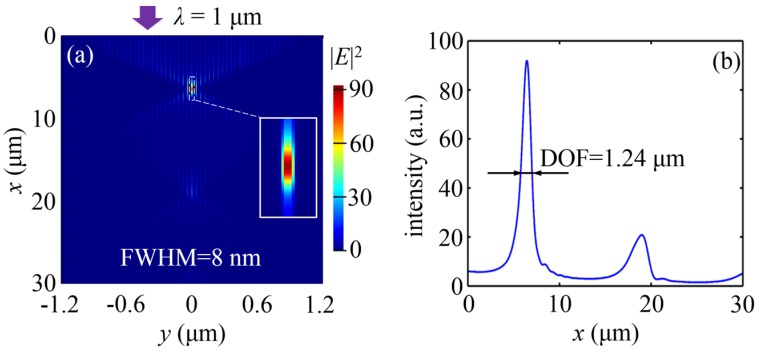
Ultra-deep sub-diffraction-limit focusing of an MGRIN lens. (**a**) FDTD-simulated electric field intensity pattern. The inset shows the enlarged view for the electric intensity distribution of the focus; (**b**) The derived |*E*|^2^ on the optical axis. The FWHM and focal depth of the focus are 8 nm and 1.24 μm, respectively.

Figure 3a presents the simulated electric field intensity distribution of the structure, which clearly confirms the focusing behavior of the MGRIN lens. The realized full width at half maximum (FWHM) at the focus is 8 nm (*λ*/125), demonstrating the ultra-deep sub-diffraction-limit focusing. This is 12.5 times smaller than the FWHM of 100 nm achieved in the previous study, using a structure with varying waveguide widths [39] for the same operating wavelength. The focal depth (DOF) is 1.24 μm (Figure 3b), and more than one wavelength, which is difficult to implement by using the previous nanofocusing approaches. Furthermore, the simulated focal length is 6.44 μm, which is close to the theoretical result of 6.87 μm calculated by the Hamiltonian optics (i.e., *π*/(2*G*)). Meanwhile, compared with the incident plane wave, the field intensity at the focus is enhanced to 92. 

**Figure 4:**
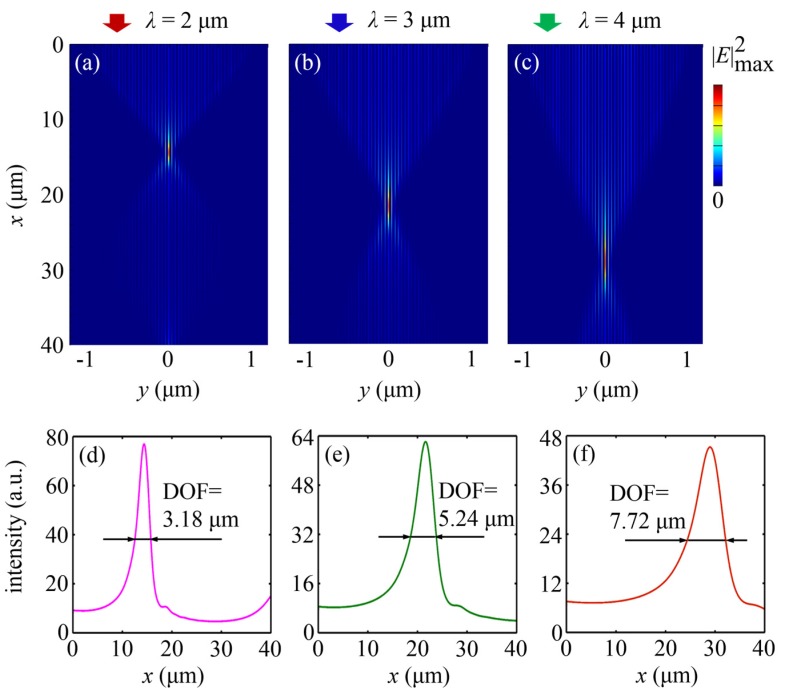
Ultra-deep sub-diffraction-limit focusing of an MGRIN lens working at longer wavelengths. (**a**–**c**) FDTD-simulated electric field intensity patterns for the wavelengths of 2–4 μm, respectively; (**d**–**f**) The corresponding |*E*|^2^ on the optical axis. The FWHMs of the three foci are all 8 nm. The focal depths at 2–4 μm are 3.18 μm, 5.24 μm, and 7.72 μm, respectively.

**Figure 5:**
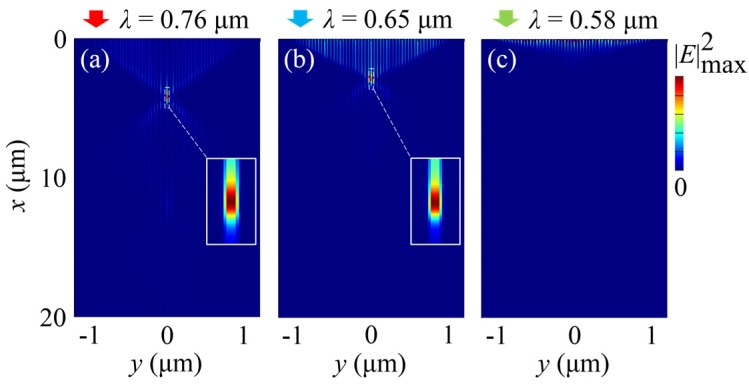
Ultra-deep sub-diffraction-limit focusing of an MGRIN lens working at the shorter wavelengths. (**a**–**c**) FDTD-simulated electric field intensity patterns for the wavelengths of 0.76 μm, 0.65 μm and 0.58 μm, respectively. The permittivity of gold at these three wavelengths is −20.273 + 0.703*i*, −12.266 + 0.779*i*, and −7.571 + 1.141*i*, respectively. The FWHMs of the two foci at 0.76 μm and 0.65 μm are both 8 nm. The corresponding focal depths are 0.86 μm and 0.88 μm, respectively.

In the previous research [39], the focusing structure needed to be redesigned when operating at a longer wavelength. In contrast, this is unnecessary for our design. We can use the same structure to obtain the nanofocusing effect for a wide range of wavelengths. Figure 4a–c show the simulated electric field intensity patterns for longer wavelengths of 2–4 μm (at 2 μm, *ε_m_* = −183.23 + 7.522*i*; at 3 μm, *ε_m_* = −415.98 + 22.462*i*; at 4 μm, *ε_m_* = −747.36 + 51.625*i*) with the same structure as the one designed for the wavelength *λ* = 1 μm. These simulation results illustrate the similar focusing behavior. For the shorter wavelengths in the visible range down to 650 nm, focusing can also be realized, as shown in Figure 5. Nevertheless, for shorter wavelengths, the nanofocusing effect cannot be observed in the structure due to the losses near the cutoff frequencies for plasma oscillations [44]. Besides the operating wavelength, the propagation losses in the structure also depend on the spacing between metallic waveguides. Losses increase with the metallic spacing. However, the metallic spacing cannot be too small to provide the capability for subwavelength optical confinement. Therefore, the metallic spacing should be appropriately selected for the nanofocusing scheme.

**Figure 6:**
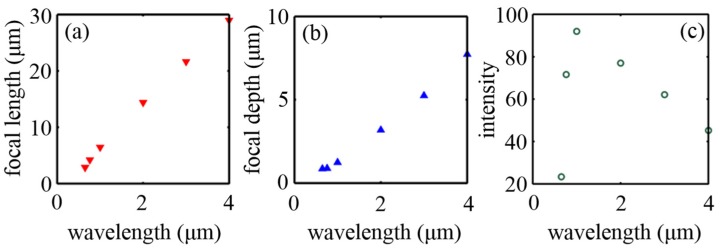
The focusing performance of an MGRIN lens working at various wavelengths from 0.65 μm to 4 μm. (**a**–**c**) Focal length, focal depth, and the maximum intensity at the focus varying as a function of the incident wavelength.

Remarkably, the FWHMs of the foci considered are all 8 nm, far beyond the diffraction limit. For the operating wavelength of 4 μm, a focus as small as *λ*/500 can be obtained. In addition, when the incident wavelength varies from 0.65 μm to 4 μm, the focal length of the MGRIN lens increases from 2.88 μm to 28.98 μm accordingly (displayed in Figure 6a). Therefore, the focal length of an MGRIN lens can be modulated by controlling the working wavelength, apart from tuning the design parameters based on Equation (2). The focal depth also increases with the wavelength (depicted in Figure 6b), which is larger than one operating wavelength. Furthermore, the field intensity enhancement for all the foci is higher than 20, as shown in Figure 6c.

**Figure 7:**
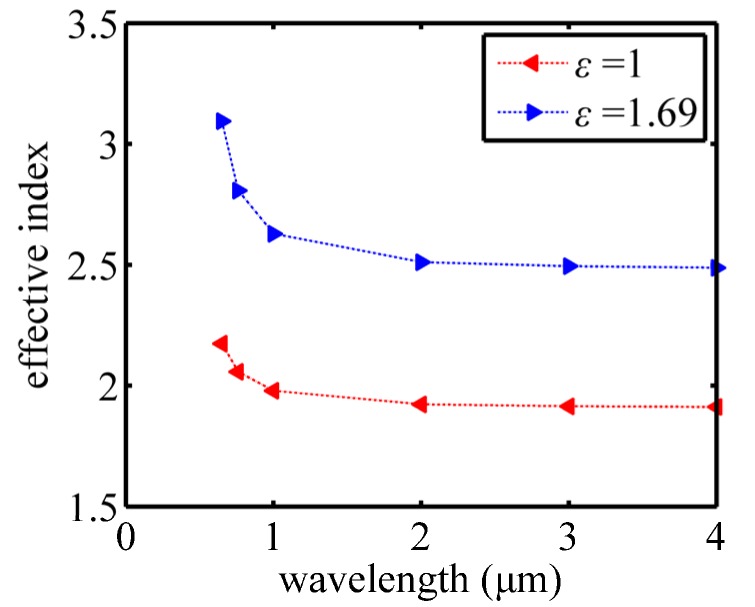
Effective indices of the central waveguide filled with a dielectric of *ε* = 1 and the side waveguide filled with a dielectric of *ε* = 1.69 for various wavelengths.

The physical mechanism behind the broadband focusing of an MGRIN lens is the small change in the effective index of a metallic waveguide. For instance, when the incident wavelength varies from 1 μm to 4 μm, the effective index of the waveguide at the side changes from 2.63 to 2.49. As for the central waveguide, the effective index change is much smaller, as shown in Figure 7. As a result, the effective indices of waveguides comprising the MGRIN lens are approximate, to meet Equation (3). This is the reason for the realization of nanofocusing with only one structure for a broad range of wavelengths.

## 4. Conclusions

In summary, based on Hamiltonian optics, a metallic graded-index (MGRIN) lens composed of an array of coupled metallic waveguides is proposed to focus light at the nanoscale. In contrast to the design of a conventional GRIN lens, the effective index for an MGRIN lens increases from the center to the side due to the negative refraction effect of coupled metallic waveguide arrays. The focusing behavior and performance of an MGRIN lens are investigated in detail via the 2D FDTD simulation method, including focal length, focal depth, and field intensity. Numerical results demonstrate that ultra-deep sub-diffraction-limit focusing (*λ*/500) with a highly enhanced field intensity and long focal depth can be achieved by an MGRIN lens. Moreover, this structure has the ability to focus over a broad range of wavelengths from visible to infrared. Such nanofocusing for broadband wavelengths could hardly be realized by previously reported methods. More importantly, the confinement capability of light based on our design can be further enhanced, provided that the metallic waveguide width can decrease further. The presented MGRIN lens shows great potential for applications in wavelength division multiplexing, nanolithography, and highly integrated optical circuits.
